# Prevalence of and risk factors for colic in horses that display crib-biting behaviour

**DOI:** 10.1186/1746-6148-10-S1-S3

**Published:** 2014-07-07

**Authors:** Ebony E Escalona, Claire N Okell, Debra C Archer

**Affiliations:** 1 Section Computational and Systems Medicine, Division of Surgery and Cancer, Faculty of Medicine, Imperial College London, Sir Alexander Fleming Building, London SW7 2AZ, UK; 2 Department of Production and Population Health, Veterinary Epidemiology, Economics and Public Health Research Group, Royal Veterinary College, Hawkshead Lane, North Mymms, AL9 7TA, UK; 3 Department of Epidemiology and Population Health, Institute of Infection and Global Health, University of Liverpool, Leahurst Campus, Wirral, CH64 7TE, UK

## Abstract

**Background:**

Crib-biting and windsucking (CBWS) behaviour in horses has been associated with increased risk of colic in general, recurrence of colic and specific forms of colic. The aims of the present study were to determine the prevalence of colic within a population of horses that display CBWS behaviour and to identify risk factors for colic.

**Methods:**

Owners/carers of horses in the general UK equine population that display CBWS behaviour were invited to participate in a questionnaire-based survey about the management and health of these horses. Data were obtained for a number of variables considered to be possible risk factors for colic. The prevalence of colic was calculated and multivariable logistic regression was used to identify associations between horse- and management-level variables for two outcomes of interest: a history of colic ever and a history of colic in the previous 12 months.

**Results:**

Data were obtained for 367 horses. One or more episodes of colic had been observed in 130 horses (35.4%). A total of 672 colic episodes were reported and 13 colic episodes required surgical intervention in 12 horses. Where the horse/pony had been in that persons care over the previous 12 months (n=331), colic had been observed in 67 horses (20.2%) during that time. A total of 126 colic episodes were reported in the preceding 12 months of which veterinary attendance was required in 69 (54.8%) episodes. Increased duration of ownership, increased duration of stabling in the Autumn months (September-November), crib-biting/windsucking behaviour associated with eating forage and horses that were fed haylage were associated with increased risk of colic (ever). Increasing severity (frequency) of CBWS behaviour and increased duration of stabling in the Autumn were associated with increased risk of colic in the previous 12 months.

**Conclusions:**

The prevalence of colic in a population of horses that display CBWS appeared to be relatively high. The results of this study can be used to identify horses that display CBWS who are at increased risk of colic and identifies areas for further research to determine if there are ways in which this risk might be reduced.

## Background

Crib-biting and windsucking are two common forms of equine stereotypic behaviour. By definition stereotypies are repetitive, relatively invariant patterns of behaviour with no apparent goal or function. They are often associated with historic or current sub-optimal environments and have been used as an indicator of welfare [1]. Crib-biting in horses is defined as the repeated seizure of fixed objects with the incisor teeth and pulling back while making a characteristic grunting noise. Windsucking is similar but the horse achieves the same posture and makes the same noise without grasping a fixed object [2] .The reported prevalence of crib-biting/windsucking behavior in captive domestic horses ranges from 2.1-10.5% [3-5] depending on the population studied. The reason why these behaviours develop in certain individuals and not in others exposed to the same environmental factors is yet to be elucidated, but is likely to be multifactorial in nature. Factors that may influence development of crib-biting/windsucking behavior include genetics, inherent differences in physiological mechanisms and various management factors, such as weaning method, diet, social contact with other horses and length of time spent outside of the stable [6].

There is evidence that crib-biting/windsucking can have detrimental effects on the health of horses that display these behaviours. These include increased prevalence of dental abnormalities [7,8], temporohyoid osteoarthropathy [9], gastric ulceration [10], poor body condition and weight loss [11] and colic [12,13]. Crib-biting/windsucking behaviour has also been identified as a risk factor for two specific forms of colic; simple colonic obstruction and distention colic [14] and epiploic foramen entrapment [15-17].

Historically, colic associated with crib-biting/windsucking behaviour was suggested to be a result of excessive ingestion of air. However, the latter theory was refuted by McGreevy et al. [8] who demonstrated that limited aerophagia actually occurred. There is increasing evidence to suggest that crib-biting/ windsucking behaviour involves a complex inter-relationship between gastrointestinal and brain function [6]. It is therefore plausible that altered intestinal and brain physiology may also play a role in the development of colic in certain individuals who display these types of stereotypic behaviour. To our knowledge no studies have investigated the prevalence of colic within a population of horses that exhibit crib-biting or windsucking behaviour. In addition, there has been no investigation of factors that increase or decrease the risk of colic occurring within this sub-population of horses.

The aims of the present study were to determine the prevalence of colic within a population of horses that display crib-biting and / or windsucking behaviour and to identify horse- and management-level risk factors for colic. *A priori* we hypothesised that factors such as type of concentrate and forage fed, length of time stabled and turned out would have an association with altered likelihood of colic.

## Methods

### Participant recruitment and data collection

Owners and carers of horses or ponies that displayed crib-biting/windsucking behaviour were recruited via adverts placed in the equine lay press, online equestrian forums and via the Philip Leverhulme Equine Hospital (University of Liverpool) website. Adverts were also emailed to yards and clubs affiliated with the British Horse Society and were given to local farriers, livery yards, veterinary surgeons and riding instructors. These adverts contained a clear description of crib-biting/ windsucking behaviour and no reference was made to any potential relationship between these forms of behaviour and colic. Participants responding to the adverts were asked to complete a postal questionnaire (see supplementary information) and return this in a prepaid return envelope. Reminder emails or postcards were sent to volunteers who had not returned a completed survey after three months and again at four months following initial questionnaire dispatch. The study was approved by the University of Liverpool Veterinary Research Ethics Committee.

The study questionnaire was constructed using information from previous epidemiological studies investigating colic [18], studies investigating equine stereotypic behaviour [5,19] and other hypotheses considered to be biologically plausible as risk factors for colic. Questions were grouped into the following categories: horse/pony signalment, use and duration of ownership; behaviour; stabling and turnout management routine; nutritional management; history of colic; routine health care; medical history; owner/carer comments on crib-biting and windsucking behaviour. Whilst we hypothesised that the prevalence of colic would be increased in these horses (compared to similar domesticated horse populations), the questionnaire was carefully constructed to minimise any form of bias e.g. ensuring there were no leading questions. Question formats included categorical choices and some open ended questions. Owners/carers were asked to grade crib-biting behaviour frequency as a categorical choice and on a 10cm (100mm) visual analogue scale (VAS). A VAS is a psychometric response scale where respondents are asked to specify their level of agreement by indicating a position along a continuous line between two end points (usually 10 cm long) [20,21]. This was scored from 0 (mild/rarely seen) to 10 (severe/seen for prolonged periods daily; see supplementary information). Data from the questionnaire were imported into a computer database using a data entry scanner (Fujitsu fi-4120C2, Fujitsu, London, UK) and Cardiff TeleForm software (Verity Inc., Illinois, USA). Scanned data were verified manually prior to committing the scanned data into the database.

### Statistical analysis

A total of 65 variables considered *a priori* as possible risk factors for colic were screened for univariable association with outcome (colic) using a Chi-squared test for categorical variables and a univariable logistic regression model for continuous variables (see supplementary information). Two outcomes were investigated: a known history of colic during ownership/care of the horse (colic ever) and a history of colic in the previous 12 months. Where variables were highly correlated (Pearson correlation coefficient >0.9) the most statistically significant or biologically plausible variable was selected. The functional form of the relationships between continuous variables and each outcome were explored using generalised additive models (GAM) [22]. Variables with *P*<0.25 were considered for inclusion in a multivariable logistic regression model which was constructed using a backwards stepwise elimination procedure. Variables remained in the model if they significantly improved the fit (*P*≤0.05), assessed using the likelihood ratio test statistic. All variables (including those with P>0.25) were then forced back into the relevant model to ensure no significant or confounding variables had been excluded. The fit of each model was also assessed using the Hosmer-Lemeshow goodness of fit test statistic. The critical probability for all analyses was set at 0.05. Data analysis was performed using Stata (Intercooled Stata 9.0, Timberlake Consultants Ltd, London, UK) and S-plus (Insightful Corp., Seattle, USA).

## Results

### Descriptive statistics

A total of 550 questionnaires were sent out to respondents, and these were based in a wide geographical area within the UK. In total 370 completed surveys were returned (response rate of 67%). Data from 367 horses were analysed; 3 horses were excluded from the present study as the horse/pony described had died several months/years previously and therefore the quality of the data was not considered to be sufficiently robust.

One or more episodes of colic had been observed in 130 horses (35.4% of horses in the study). A total of 672 colic episodes were reported by the owners/carers of these horses (range 1-50 colic episodes; mean 5.25). Thirteen colic episodes were reported to have required surgical intervention in 12 horses (1.9% of all colic episodes); it was not possible to determine reliably from the owner/carer the specific cause of colic in these cases. Where the horse/pony had been in that persons care over the previous 12 months (n=331), colic had been observed in 67 horses (20.2%) during that time. A total of 126 colic episodes were reported in the preceding 12 months (range 1-8, mean 1.88) of which veterinary attendance was required in 69 (54.8%) episodes. This equates to 38 episodes of colic per 100 horse years at risk (HYAR) for all colic episodes or 20 veterinary attended episodes of colic per 100 HYAR.

### Univariable analysis

**Figure 1:**
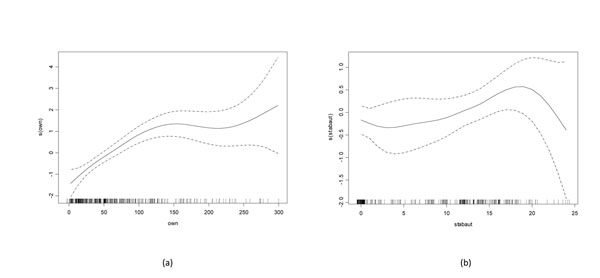
**Generalised additive models describing the functional form of the relationship between duration of ownership and hours stabled and the log odds of having a history of previous colic (ever)** Use of generalised additive models to describe the functional form of the relationship between (a) duration of ownership and (b) hours stabled in the autumn months with the outcome (log odds of having ever suffered from colic). The plots show the fitted curves with 95% confidence intervals (dashed lines) and the rug plots along the x axis represent the number of data points.

**Figure 2:**
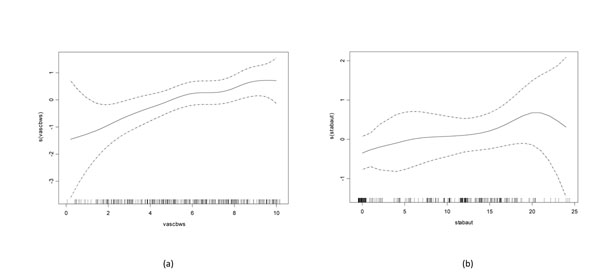
**Generalised additive models describing the functional form of the relationship between severity of crib-biting/windsucking behaviour and hours stabled in the Autumn and the log odds of having a history of colic in the previous 12 months** Use of generalised additive models to describe the functional form of the relationship between (a) severity of cribbiting/windsucking behaviour and (b) hours stabled in the autumn months with the outcome (log odds of having suffered from colic in the previous 12 months). The plots show the fitted curves with 95% confidence intervals (dashed lines) and the rug plots along the x axis represent the number of data points.

The results of univariable screening for variables with a P<0.25 for each outcome of interested are provided in the supplementary information (Additional files 1, 2, 3, 4). Examination of generalised additive model plots for continuous variables significantly associated with altered risk of colic in both models indicated that a linear fit was appropriate for each (Figures 1 and 2).

### Multivariable analysis

Final multivariable models for each outcome investigated (colic history ever and history of colic in the previous 12 months) are shown in Table 1. In Model 1, increased likelihood of colic (ever) was associated with increased duration of ownership and increased duration of stabling during the Autumn months (September-November). Horses who owners’/carers’ reported that crib-biting/windsucking behaviour was exhibited more frequently in association with eating forage were around twice as likely to have a history of colic as were horses that received haylage as a source of forage. In Model 2, increased hours stabled during the Autumn months were significantly associated with increased likelihood of a history of colic within the previous 12 months as was increased severity of crib-biting / windsucking behaviour as perceived by owners/carers. No significant biologically plausible multiplicative interactions were found between the variables in the final models. The Hosmer-Lemeshow test statistic indicated no evidence of poor fit in either model (Model 1 P=0.34, Model 2 P=0.55).

**Table 1:** Multivariable logistic regression model of risk factors for colic in a population of horses that exhibit crib-biting / windsucking behaviour.

Variable		Coefficient	Standard error	Adjusted odds ratio	95% Confidence Interval	Likelihood ratio P value
**Model 1: History of colic ever**						

Duration of ownership (months)		0.016	0.003	1.02	1.01 - 1.02	<0.001
Duration of stabling in the Autumn (hours per day)		0.041	0.019	1.04	1.003 - 1.08	0.035
CBWS behaviour associated with eating forage	No			Ref.		
	Yes	0.773	0.279	2.17	1.25 – 3.74	0.006
Fed haylage	No			Ref.		
	Yes	0.733	0.279	2.08	1.20 – 3.60	0.008

**Model 2: History of colic in the last 12 months**						

Severity of CBWS behaviour (cm)		0.217	0.059	1.24	1.10 - 1.40	<0.001
Duration of stabling in the Autumn (hours per day)		0.050	0.021	1.05	1.01 – 1.10	0.016

The outcome of interest is colic ever in Model 1 and colic within the previous 12 months in Model 2.

## Discussion

The present study has provided information about the prevalence of colic in a population of UK horses that display crib-biting / windsucking behaviour. In addition, we have identified a number of horse- and management-level risk factors that are associated with increased likelihood of a history of colic in horses that display these forms of stereotypic behaviour.

The prevalence of colic within a UK population horses that display crib-biting/windsucking behaviour appeared to be high (38 colic episodes per 100 HYAR for all colic episodes and 20 veterinary attended episodes of colic per 100 HYAR), and a proportion of these horses were reported to have had multiple recurrences of colic. Within the general (managed) equine population, the prevalence of colic in published studies varies from 3.5-10.6 colic episodes per 100 horses per year (i.e. per 100 HYAR) [23-26] which is lower than the present study. It is important to note that, because these populations may differ from those investigated in the present study (e.g. geography and population demographics), and because this study did not measure prevalence of colic in a similar population of horses that did not display crib-biting/windsucking behaviour, direct comparisons between these studies cannot be reliably made. In a study investigating risk factors for epiploic foramen entrapment colic in the UK [16] in a similar, predominantly pleasure horse population, of 522 randomly selected control horses (i.e. non-EFE horses randomly selected from the same population), 41 horses (7.8%) were reported to have had one or more episodes of colic (veterinary and non-veterinary attended) in the 12 months prior to the questionnaire being administered. A total of 80 colic episodes were reported, equating to 15.3 episodes of colic per 100 HYAR (previously unpublished data), which is again less than the prevalence found in the present study. The apparent high prevalence of colic in the present study is in agreement with a study conducted by Malamed et al. [11] who reported that horses that had a history of crib-biting or windsucking behaviour were twice as likely to have a history of previous colic compared to those that did not display these stereotypies. However, the latter study was undertaken in a population of horses referred to an equine clinic which may reflect a biased population. Recurrence of colic was also common in a sub-group of these horses in the present study. This is in agreement with the findings of Scantlebury et al. [13] who found that horses that displayed crib-biting/windsucking behaviour were 12 times more likely to have a history of recurrent colic compared to horses that did not display these behaviours. Most colic episodes reported in the present study were medical in nature and very few of these episodes necessitated surgical intervention (1.9%). We do not suggest that crib-biting/windsucking behaviour causes colic, and the majority of horses in the present study had no prior history of colic. However, it is possible that a sub-population of horses that display crib-biting/windsucking behaviour exists that may have some inherent difference in intestinal function that is associated (potentially causally with respect to colic) with both CBWS and colic.

Owner/carer perceived severity of crib-biting/windsucking behaviour as assessed using a VAS was significantly associated with altered likelihood of colic in the previous 12 months. VAS have a number of applications in clinical situations including behaviour-based assessment of colic pain [25] or level of sedation, analgesia and behaviour during standing surgery [19] in horses. Horses that were considered to display this behaviour with increased frequency (severity) were at significantly increased risk of colic. This outcome was also significantly associated with the categorical measure of crib-biting/windsucking frequency but as both variables were highly correlated, the VAS score was chosen as it fitted the model best. Altered gastrointestinal function is hypothesised to play an important role in development of crib-biting/windsucking behaviour [6]. The results of the present study may suggest that, if there are differences in the inherent and complex inter-relationship between brain and gut function, these differences may also vary between horses that display crib-biting/behaviour. This could be a plausible explanation for why horses with most marked expressions of these forms of behaviour are also at significantly increased risk of colic and this hypothesis merits further research. It is also possible that the observed association might be due to selection and recall bias if respondents held a prior belief of some association between crib-biting/windsucking and colic.

Increased duration of ownership or care of horses was associated with increased likelihood of a history of colic (ever). This would appear to reflect increased opportunity for horses to suffer from colic rather than any direct effect on colic risk. However, this variable was considered to be a plausible factor in altered risk of colic (e.g. a horse that has only been in the owners care a short time may be at increased risk of colic) and is the reason why it was considered to be worthy of investigation. Increasing age did not have any significant effect on likelihood of colic nor did the number of carers or time spent with horses on a daily basis.

Horses that displayed increased frequency of crib-biting/windsucking behaviour when eating forage (such as hay or haylage) were around twice as likely to have a history of colic (ever) compared to those in which behaviour patterns remained unchanged. A number of studies have demonstrated an association between increased expression of crib-biting/windsucking behaviour and feeding of concentrates [6] and we wished to explore whether concentrate feeding and its associations with these forms of behaviour had any association with risk of colic too. In most horses in the present study, this association was noted in these horses by their owners/carers but it had no association with likelihood of colic. Lack of dietary forage is a factor implicated in development of crib-biting/windsucking behaviour and provision of sufficient forage together with opportunities for horses to display normal foraging behaviour are recommended as ways in which crib-biting/windsucking behaviour may be attenuated [27]. If expression of crib-biting/windsucking behaviour and increased risk of colic is somehow linked, then provision of forage, which should reduce expression of crib-biting behaviour, might be expected to reduce the risk of colic developing. Therefore the finding that horses considered by their owner/carer to demonstrate increased expression of crib-biting/windsucking behaviour when eating forage had a significantly increased risk of colic might appear counter-intuitive. This finding merits further investigation to determine whether a sub-population of horses that display crib-biting/windsucking behaviour exhibit altered physiological response to forage feeding and if such a difference exists, whether this is also associated with increased risk of colic in horses.

In the present study, horses that were fed haylage were also twice as likely to have a history of colic compared to those that did not receive this form of forage. Provision of hay and specific types of hay have previously been identified to alter the risk of colic and increase the likelihood of specific forms of colic such as ileal impactions [28] and large colon volvulus [29]. Haylage has not to the authors’ knowledge been identified as a risk factor for colic in previous studies. This may be in part due to the specific type of forage provided not always being separated into hay, haylage or other forms of conserved forage in other owner questionnaire based studies and it is unknown whether this association may exist in a similar population of horses that do not exhibit crib-biting/windsucking behaviour. The results of the present study would suggest that the risk of colic in horses that crib-bite/windsuck could potentially be reduced if fed other forms of forage. However, the chemical composition of haylage can vary widely due to a number of factors such as grass species, maturity of plants at the time of harvesting and the degree of fermentation or spoilage that may occur [30,31]. Therefore further investigations should determine the specific nutrient values of forages provided, including those in haylage and association with altered likelihood of colic.

Increased duration of stabling in the autumn months (September–November) was associated with both increased likelihood of a history of colic ever and increased likelihood of colic within the previous 12 months. Increasing time spent stabled and reduced time spent out at pasture have been identified as factors that increase the risk of colic in previous studies [14,16,29,32]. Whilst duration of time spent stabled at other times of the year were not significantly associated with risk of colic, the autumn months would generally be considered to be a time when management changes such as feeding and stabling change in the UK, dependent on a number of factors such as prevailing weather conditions and pasture available for grazing. Colic has been previously identified to have a seasonal pattern, with colic in general being more common in the spring and autumn months [33]. It was not possible to obtain sufficiently accurate data about seasonal patterns in the present study to determine if there was any seasonal pattern of colic in this population of horses and whether these patterns mirrored management changes including duration of stabling.

The present study was part of a questionnaire survey about the general health and management of horses that display crib-biting/ windsucking behaviour. Response rates were comparable to other questionnaire studies mailed to horse owners in the UK [34]. It is always important to consider possible biases that may arise in observational studies such as this. The design of this study may have led to inherent selection bias due to only soliciting answers from owners of horses who display crib-biting/winduscking behaviour (rather than the general equine population) and it is possible that further selection bias may have occurred as data from non-respondents could not be measured. Particular care was taken in the recruitment of participants and in the design of the questionnaire to try to avoid any suggestion (or otherwise) of any association between these forms of behaviour and colic (as the prevalence of colic in these horses was something we wished to measure). Despite this, it is possible that respondents may have been biased towards those whose horses had prior or ongoing health issues such as colic and/or respondents who had any prior beliefs about crib-biting/windsucking behaviour and colic. As with all studies investigating historic health and management details it is possible that there may have been some recall bias [35], as recollection of events is likely to be less accurate than data obtained from logs or medical charts. In addition if respondents held prior beliefs about an association between crib-biting/windsucking behaviour and colic, they may have been more likely to recall prior colic episodes. Misclassification bias may also have occurred if a colic episode was incorrectly diagnosed by the owner/carer. However, colic is perceived by owners/carers as a serious health condition [34] and it is likely that episodes that occurred would be noted and remembered by owners. Where horses had a history of one of more colic episodes in the previous 12 months, most episodes were veterinary attended, and colic was confirmed as a diagnosis by the attending veterinary surgeon. This together with the fact that most horse owners are aware of the signs of colic should minimise the possibility that a colic episode was incorrectly diagnosed by the owner/carer.

Many owners/carers attempt to physically prevent horses from performing crib-biting behaviour [3] but these methods fail to address the underlying cause of these behaviours and may further reduce equine welfare [6]. Participants were asked if they had used any method to prevent crib-biting/windsucking (e.g. collars and stable toys), but the question was not phrased appropriately to determine if these measures had any effect on these behaviours or the likelihood of colic. We did not specifically ask when crib-biting/windsucking behaviour started, as in one of the author’s experience (DCA) when discussing this form of behaviour in horses with owners/carers, they frequently report that this behaviour was exhibited at the time of purchase and the horse’s prior history, including the age at which this behaviour started, is often unknown. Some investigators consider that crib-biting/windsucking behaviour is exhibited as a response to some form of abdominal pain [6] but we did not explore whether there was any temporal relationship between crib-biting/windsucking behaviour and colic in the present study.

Therefore there is a need for a prospective longitudinal study investigating risk of colic in a cohort of horses that display crib-biting/windsucking behaviour where more detailed information about exposures such as diet can be more accurately measured. This could also enable further investigation of seasonal patterns in occurrence of colic, including the effect of factors such as feeding practices and stabling/turnout, and possible temporal relationships between crib-biting/windsucking behaviour and risk of colic.

## Conclusions

The proportion of horses that had a previous history of colic and the prevalence of colic in a population of horses that exhibit crib-biting/windsucking behaviour appeared to be high in the present study. Increased likelihood of colic was associated with increased severity of crib-biting/windsucking behaviour as assessed by the owner/carer, increased duration of stabling in the autumn months, feeding of haylage and individuals in which eating forage was associated with increased expression of crib-biting/windsucking behaviour. This study has highlighted areas that require further research, particularly in relation to nutrition and the risk of colic. Knowledge of these risk factors may assist in identifying crib-biting/windsucking horses that are at increased risk of colic and managemental practices that should be investigated further to determine whether these risks are alterable.

## Competing interests

The authors declare that they have no competing interests.

## Authors' contributions

EE designed the questionnaire, recruited participants, acquired the data, performed preliminary statistical analysis and helped to draft the manuscript. CO assisted with preliminary data analysis and writing of the manuscript. DA conceived of the study, assisted design of the questionnaire, performed final statistical analysis and drafted the manuscript. All authors have read and approved the final manuscript.

## Supplementary Material

Additional file 1 **Univariable analysis of categorical variables and their relationship with likelihood of a history of colic (ever)** Univariable analysis of categorical variables investigated for association with a history of colic ever in a population of 367 horses / ponies that display crib-biting / windsucking behaviour with P<0.25. CI=confidence interval, Tb= Thoroughbred, ISH= Irish Sports Horse, Wb=Warmblood. Click here for file

Additional file 2 **Univariable logistic regression analyses of continuous variables and their relationship with the likelihood of a history of colic (ever)** Descriptive statistics and univariable logistic regression analysis of continuous variables investigated for association with a history of colic ever in 367 horses with P<0.25. CI = Confidence Interval, VAS= Visual Analogue Scale. Click here for file

Additional file 3 **Univariable analysis of categorical variables and their relationship with likelihood of a history of colic (ever)** Univariable analysis of categorical variables investigated for association with a history of colic in the previous 12 months in a population of horses that display crib-biting / windsucking behaviour with P<0.25. CI=confidence interval, Tb= Thoroughbred, ISH= Irish Sports Horse, Wb=Warmblood. Click here for file

Additional file 4 **Univariable logistic regression analyses of continuous variables and their relationship with the likelihood of a history of colic (in the previous 12 months)** Descriptive statistics and univariable logistic regression analysis of continuous variables investigated for association with a history of colic in the previous 12 months with P<0.25. CI= Confidence Interval, VAS= Visual Analogue Scale. Click here for file

## Declarations

This article has been published as part of *BMC Veterinary Research* Volume 10 Supplement 1, 2014: Selected articles from the Eleventh International Equine Colic Research Symposium. The full contents of the supplement are available online at http://www.biomedcentral.com/bmcvetres/supplements/10/S1. Publication of this supplement has been funded by The British Equine Veterinary Association.
