# Supplementary material for: Prevalence of and risk factors for colic in horses that display crib-biting behaviour
Source: BMC Vet Res. 2014 Jul 7;10(Suppl 1):S3. doi: 10.1186/1746-6148-10-S1-S3 (PMC4123051; doi:10.1186/1746-6148-10-S1-S3)
Supplement: Additional file 1 — Univariable analysis of categorical variables and their relationship with likelihood of a history of colic (ever) Univariable analysis of categorical variables investigated for association with a history of colic ever in a population of 367 horses / ponies that display crib-biting / windsucking behaviour with P<0.25. CI=confidence interval, Tb= Thoroughbred, ISH= Irish Sports Horse, Wb=Warmblood. [file 1746-6148-10-S1-S3-S1.docx]

**Additional file 1.** Univariable analysis of categorical variables investigated for association with a history of colic ever in a population of 367 horses / ponies that display crib-biting / windsucking behaviour with P<0.25. CI=confidence interval, Tb= Thoroughbred, ISH= Irish Sports Horse, Wb=Warmblood.

| **Variable** | **Coefficient** | **Standard Error** | **Odds ratio** | **95% CI** | **P value** |
| --- | --- | --- | --- | --- | --- |
| **General use** |  |  |  |  |  |
| No |  |  | Ref. |  |  |
| Yes | 0.53 | 0.23 | 1.70 | 1.08-2.67 | 0.02 |
| **Breeding use** |  |  |  |  |  |
| No |  |  | Ref. |  |  |
| Yes | -0.939 | 0.508 | 0.39 | 0.14-1.06 | 0.04 |
| **Being broken in** |  |  |  |  |  |
| No |  |  | Ref. |  |  |
| Yes | -1.391 | 0.764 | 0.25 | 0.06-1.11 | 0.03 |
| **Crib-biting/windsucking behaviour demonstrated when in a stable** |  |  |  |  |  |
| No |  |  | Ref. |  |  |
| Yes | 1.120 | 0.639 | 3.06 | 0.88-10.72 | 0.05 |
| **Crib-biting/windsucking behaviour demonstrated when turned out in a field** |  |  |  |  |  |
| No |  |  | Ref. |  |  |
| Yes | 0.623 | 0.285 | 1.87 | 1.07-3.26 | 0.02 |
| **Frequency of crib-biting / windsucking behaviour** |  |  |  |  |  |
| Seen weekly or less but not everyday / only during specific situations |  |  | Ref. |  |  |
| Seen everyday for short periods of time | 0.941 | 0.392 | 2.56 | 1.19-5.52 | 0.016 |
| Seen everyday for prolonged periods of time | 1.460 | 0.434 | 4.31 | 1.84-10.08 |  |
| **Frequency of crib-biting /windsucking behaviour in relation to eating concentrate (hard) feed** |  |  |  |  |  |
| No increase / same frequency with feeding this |  |  | Ref. |  |  |
| Increased frequency with feeding of this | 1.215 | 0.403 | 3.37 | 1.53-7.42 | 0.001 |
| **Frequency of crib-biting /windsucking behaviour in relation to eating forage feed** |  |  |  |  |  |
| No increase / same frequency with feeding this |  |  | Ref. |  |  |
| Increased with feeding this | 0.662 | 0.20-1.12 | 1.94 | 1.22-3.07 | 0.004 |
| **Premises type livery yard** |  |  |  |  |  |
| No |  |  | Ref. |  |  |
| Yes | 0.462 | 0.350 | 1.59 | 0.80-3.15 | 0.19 |
| **Turnout on grass** |  |  |  |  |  |
| No |  |  | Ref. |  |  |
| Yes | -0.646 | 0.396 | 0.52 | 0.24-1.14 | 0.10 |
| **Forage type – hay** |  |  |  |  |  |
| No |  |  | Ref. |  |  |
| Yes | -0.287 | 0.230 | 0.75 | 0.48-1.18 | 0.21 |
| **Forage type - haylage** |  |  |  |  |  |
| No |  |  | Ref. |  |  |
| Yes | 0.390 | 0.231 | 1.48 | 0.94-2.32 | 0.09 |
| **Frequency of feeding concentrate feed** |  |  |  |  |  |
| None / once daily |  |  | Ref. |  |  |
| Twice daily | 0.789 | 0.255 | 2.20 | 1.33-3.63 | 0.004 |
| Three times daily | 0.154 | 0.494 | 1.17 | 0.44-3.07 |  |
