# Supplementary material for: Prevalence of and risk factors for colic in horses that display crib-biting behaviour
Source: BMC Vet Res. 2014 Jul 7;10(Suppl 1):S3. doi: 10.1186/1746-6148-10-S1-S3 (PMC4123051; doi:10.1186/1746-6148-10-S1-S3)
Supplement: Additional file 2 — Univariable logistic regression analyses of continuous variables and their relationship with the likelihood of a history of colic (ever) Descriptive statistics and univariable logistic regression analysis of continuous variables investigated for association with a history of colic ever in 367 horses with P<0.25. CI = Confidence Interval, VAS= Visual Analogue Scale. [file 1746-6148-10-S1-S3-S2.docx]

**Additional file 2**. Descriptive statistics and univariable logistic regression analysis of continuous variables investigated for association with a history of colic ever in 367 horses with P<0.25. CI = Confidence Interval, VAS= Visual Analogue Scale.

| **Variable** | **Mean / Median** | **Coefficient** | **Standard error** | **Odds ratio** | **95% CI** | **P value** |
| --- | --- | --- | --- | --- | --- | --- |
| Age (years) | 12.01 | 0.11 | 0.02 | 1.11 | 1.07-1.16 | <0.001 |
| Duration of ownership (months) | 68.60 | 0.01 | 0.002 | 1.01 | 1.01-1.02 | <0.001 |
| Height (cm) | 159.67 | 0.02 | 0.01 | 1.02 | 1.004-1.05 | 0.01 |
| Owner perception of severity of crib-biting / windsucking behaviour (VAS scale 0-10) | 5.60 | 0.14 | 0.04 | 1.15 | 1.05-1.25 | 0.002 |
| Number of horses on the premises | 18.95 | -0.006 | 0.004 | 0.99 | 0.99-1.003 | 0.14 |
| Number of carers | 1.99 | -0.08 | 0.08 | 0.92 | 0.79-1.08 | 0.27 |
| Hours stabled in the spring months (March-May) (hours/day) | 9.14 | 0.02 | 0.02 | 1.02 | 0.99-1.05 | 0.25 |
| Hours stabled in the autumn months (September-November) (hours/day) | 8.20 | 0.03 | 0.02 | 1.03 | 0.99-1.06 | 0.07 |
| Hours turned out in the spring months (March-May) (hours/day) | 14.51 | -0.02 | 0.01 | 0.98 | 0.95-1.007 | 0.14 |
| Hours turned out in the summer months (June-August) (hours / day) | 18.70 | -0.02 | 0.02 | 0.98 | 0.95-1.01 | 0.21 |
| Hours turned out in the autumn months (September-November) (hours/day) | 15.50 | -0.02 | 0.01 | 0.98 | 0.95-1.01 | 0.12 |
