# Supplementary material for: Prevalence of and risk factors for colic in horses that display crib-biting behaviour
Source: BMC Vet Res. 2014 Jul 7;10(Suppl 1):S3. doi: 10.1186/1746-6148-10-S1-S3 (PMC4123051; doi:10.1186/1746-6148-10-S1-S3)
Supplement: Additional file 3 — Univariable analysis of categorical variables and their relationship with likelihood of a history of colic (ever) Univariable analysis of categorical variables investigated for association with a history of colic in the previous 12 months in a population of horses that display crib-biting / windsucking behaviour with P<0.25. CI=confidence interval, Tb= Thoroughbred, ISH= Irish Sports Horse, Wb=Warmblood. [file 1746-6148-10-S1-S3-S3.docx]

**Additional file 3.** Univariable analysis of categorical variables investigated for association with a history of colic in the previous 12 months in a population of horses that display crib-biting / windsucking behaviour with P<0.25. CI=confidence interval, Tb= Thoroughbred, ISH= Irish Sports Horse, Wb=Warmblood.

| **Variable** | **Coefficient** | **Standard Error** | **Odds ratio** | **95% CI** | **P value** |
| --- | --- | --- | --- | --- | --- |
| **Arab breed** |  |  |  |  |  |
| No |  |  | Ref. |  | 0.09 |
| Yes | 1.21 | 0.695 | 3.35 | 0.87-12.86 |  |
| **Body condition score** |  |  |  |  |  |
| Normal / overweight (BCS 3/4) |  |  | Ref. |  | 0.15 |
| Lean / very lean (BCS 1/2) | 0.505 | 0.341 | 1.66 | 0.85-3.22 |  |
| **General use** |  |  |  |  |  |
| No |  |  | Ref. |  | 0.17 |
| Yes | 0.394 | 0.292 | 1.48 | 0.84-2.63 |  |
| **Eventing use** |  |  |  |  |  |
| No |  |  | Ref. |  |  |
| Yes | -0.637 | 0.371 | 0.53 | 0.26-1.09 | 0.07 |
| **Riding/pony club use** |  |  |  |  |  |
| No |  |  | Ref. |  |  |
| Yes | -0.402 | 0.330 | 0.67 | 0.35-1.28 | 0.21 |
| **Crib-biting/windsucking behaviour demonstrated when in a stable** |  |  |  |  |  |
| No |  |  | Ref. |  |  |
| Yes | 1.509 | 1.038 | 4.52 | 0.59-34.6 | 0.07 |
| **Frequency of crib-biting / windsucking behaviour** |  |  |  |  |  |
| Seen weekly or less but not everyday / only during specific situations |  |  | Ref. |  |  |
| Seen everyday for short periods of time | 0.874 | 0.552 | 2.39 | 0.81-7.06 | 0.03 |
| Seen everyday for prolonged periods of time | 1.423 | 0.589 | 4.14 | 1.31-13.16 |  |
| **Frequency of crib-biting /windsucking behaviour in relation to feeding concentrate (hard) feed** |  |  |  |  |  |
| No increase / same frequency with feeding this |  |  | Ref. |  |  |
| Increased frequency with feeding of this | 0.835 | 0.495 | 2.30 | 0.87-6.09 | 0.07 |
| **Frequency of crib-biting /windsucking behaviour in relation to eating forage feed** |  |  |  |  |  |
| No increase / same frequency when eating forage |  |  | Ref. |  |  |
| Increased frequency when eating forage | 0.372 | 0.288 | 1.13 | 0.93-2.03 | 0.19 |
| **Premises type livery yard** |  |  |  |  |  |
| No |  |  | Ref. |  |  |
| Yes | 0.908 | 0.378 | 2.48 | 1.18-5.21 | 0.02 |
