# Supplementary material for: Prevalence of and risk factors for colic in horses that display crib-biting behaviour
Source: BMC Vet Res. 2014 Jul 7;10(Suppl 1):S3. doi: 10.1186/1746-6148-10-S1-S3 (PMC4123051; doi:10.1186/1746-6148-10-S1-S3)
Supplement: Additional file 4 — Univariable logistic regression analyses of continuous variables and their relationship with the likelihood of a history of colic (in the previous 12 months) Descriptive statistics and univariable logistic regression analysis of continuous variables investigated for association with a history of colic in the previous 12 months with P<0.25. CI= Confidence Interval, VAS= Visual Analogue Scale. [file 1746-6148-10-S1-S3-S4.docx]

**Additional file 4.** Descriptive statistics and univariable logistic regression analysis of continuous variables investigated for association with a history of colic in the previous 12 months with P<0.25. CI= Confidence Interval, VAS= Visual Analogue Scale.

| **Variable** | **Mean / Median** | **Coefficient** | **Standard error** | **Odds ratio** | **95% CI** | **P value** |
| --- | --- | --- | --- | --- | --- | --- |
| Age (years) | 12.4 | 0.041 | 0.022 | 1.04 | 0.99-1.08 | 0.07 |
| Duration of ownership (months) | 74.5 | 0.04 | 0.002 | 1.004 | 1.0001-1.008 | 0.048 |
| Owner perception of severity of crib-biting / windsucking behaviour (VAS scale) | 5.6 | 0.200 | 0.058 | 1.22 | 1.09-1.37 | 0.0003 |
| Hours stabled in the spring months (March-May) (hours/day) | 9.41 | 0.023 | 0.019 | 1.02 | 0.98-1.06 | 0.24 |
| Hours stabled in the summer months (June-August) (hours / day) | 5.09 | 0.027 | 0.021 | 1.02 | 0.99-1.07 | 0.20 |
| Hours stabled in the autumn months (September-November) (hours/day) | 8.27 | 0.040 | 0.020 | 1.04 | 1.0007-1.08 | 0.04 |
| Number of carers | 2.01 | -0.208 | 0.142 | 0.81 | 0.61-1.07 | 0.08 |
| Hours turned out in the summer months (June-August) (hours / day) | 18.5 | -0.025 | 0.019 | 0.97 | 0.94-1.01 | 0.21 |
| Hours turned out in the autumn months (September-November) (hours/day) | 15.4 | -0.034 | 0.019 | 0.97 | 0.93-1.003 | 0.07 |
